# Joule Heating-Induced Carbon Fibers for Flexible Fiber Supercapacitor Electrodes

**DOI:** 10.3390/ma13225255

**Published:** 2020-11-20

**Authors:** Jin Gu Kang, Gang Wang, Sung-Kon Kim

**Affiliations:** 1 Nanophotonics Research Center, Korea Institute of Science and Technology, Seoul 02792, Korea; lucid1@kist.re.kr; 2 Beckman Institute for Advanced Science and Technology, University of Illinois at Urbana-Champaign, Urbana, IL 61801, USA; gangwang@illinois.edu; 3 School of Chemical Engineering, Jeonbuk National University, 567 Baekje-daero, Deokjin-gu, Jeonju-si, Jeollabuk-do 54896, Korea

**Keywords:** supercapacitor, fiber electrode, Joule heating, energy storage, wearable device

## Abstract

Microscale fiber-based supercapacitors have become increasingly important for the needs of flexible, wearable, and lightweight portable electronics. Fiber electrodes without pre-existing cores enable a wider selection of materials and geometries than is possible through core-containing electrodes. The carbonization of fibrous precursors using an electrically driven route, different from a conventional high-temperature process, is particularly promising for achieving this structure. Here, we present a facile and low-cost process for producing high-performance microfiber supercapacitor electrodes based on carbonaceous materials without cores. Fibrous carbon nanotubes-agarose composite hydrogels, formed by an extrusion process, are converted to a composite fiber consisting of carbon nanotubes (CNTs) surrounded by an amorphous carbon (aC) matrix via Joule heating. When assembled into symmetrical two-electrode cells, the composite fiber (aC-CNTs) supercapacitor electrodes deliver a volumetric capacitance of 5.1 F cm^−3^ even at a high current density of 118 mA cm^−3^. Based on electrochemical impedance spectroscopy analysis, it is revealed that high electrochemical properties are attributed to fast response kinetics with a characteristic time constant of 2.5 s. The aC-CNTs fiber electrodes exhibit a 94% capacitance retention at 14 mA cm^−3^ for at least 10,000 charge-discharge cycles even when deformed (90° bend), which is essentially the same as that (96%) when not deformed. The aC-CNTs fiber electrodes also demonstrate excellent storage performance under mechanical deformation—for example, 1000 bending-straightening cycles.

## 1. Introduction

The continuing progress in portable microelectronics has triggered an explosion of research into energy storage devices with flexible, wearable, and lightweight features which can fit curved and complex geometries [1,2,3]. Fiber supercapacitors (fSCs) at the microscale are one class of electrochemical energy storage devices that fulfill these requirements. They are typically composed of a pair of fibrous electrodes with a diameter in the microscale separated by the solid-state electrolyte. At the interface between the electrode and the electrolyte, the electrode stores and frees charges based on physical interactions or redox reactions [4]. Recent investigations have demonstrated that fSCs can be successfully integrated into complicated geometries with limited space, such as textiles and microscale devices, while preserving a high power density and a long cycle life [5,6,7,8,9,10,11,12,13,14,15,16,17,18,19,20,21]. Electrode materials used for fSCs typically include carbonaceous materials [5,6,7,8,9,10,11,12], metal oxides [13,14,15,16,17], and conductive polymers [18,19,20,21]. Of all these, carbonaceous materials show promise as they generally demonstrate a remarkably high power density and a long cycle life. It is well known that this is possible because they reversibly accumulate and release charge via the physical adsorption or desorption of ions at the electrical double layer, formed at the interfaces between the electrode and the electrolyte, without accompanying redox reactions [4].

One popular way of producing carbonaceous materials is carbonization, a thermally activated, oxygen-free process [22]. The precursor type and temperature are major factors in determining the final products; amorphous, graphitic, or mixed-phase carbons [22,23,24]. Many fSC electrodes have been fabricated by the carbonization of pre-existing solid core-fibers, including yarn [8,25], cellulose [26], nanofiber [27], silk [28], and fabrics [29]; however, they have a limited geometry and selection of starting materials for carbonaceous materials. Therefore, the carbonization of precursors not containing core-fibers may provide a higher degree of freedom in the choice of components and geometries. Hydrogels or viscous liquids with deformable but not flowing features are particularly promising as initial precursors of core-free carbonaceous fibers. It may be possible to achieve more complex geometries other than curved, twisted, and knitted ones because of their form factor flexibility; however, carbonized fiber electrodes based on this approach are very rare [30].

One important aspect that needs to be considered the most when carbonizing microscale fibrous hydrogels is how to homogeneously transform the entire sample to carbon while retaining their original fibrous morphology. Conventional thermal carbonization is not a suitable approach because it involves a high-temperature (typically, 500–1000 °C) process for several hours, enough to distort the fiber structure of hydrogels and high-viscosity liquids. One alternative route could be hydrothermal carbonization, which adopts a low-temperature process (150–200 °C) [31]; however, fibrous hydrogels were deformed and contaminated during carbonization in a liquid environment combined with high-pressure conditions. Laser-induced carbonization is an emerging technique, enabling the carbonization of micro-patterned samples [32]; however, it requires point-to-point scanning with a tightly focused laser beam, which is time consuming, costly, and may cause nonuniform heating unless laser scanning is very precisely controlled. As an alternative, a nanosoldering technique has recently proved effective in concurrently achieving the carbonization of poly(vinyl alcohol) (PVA) and the chemical crosslinking of embedded carbon nanotubes (CNTs) [33]. Unlike conventional carbonization, this method utilizes Joule heating, generated by applying a high voltage across both ends of a fiber under vacuum, yielding carbon phases from the decomposition of precursors. The fiber supercapacitor (SC) electrodes produced from this nanosoldering process demonstrate a remarkably high volumetric energy density and stable electrochemical performances when subjected to bending, which is attributed to the CNTs that are tightly linked through covalent bonds. However, the possibility of generalizing this approach to produce core-free fiber composite electrodes still remains unexplored.

Here, using the nanosoldering technique, we fabricate highly flexible, core-fiber-free composite fSCs electrodes consisting of CNTs homogeneously embedded in an amorphous carbon matrix. Agarose and CNTs were used as the starting materials of composite fibers. Agarose is a hydrophilic natural polymer and forms a highly deformable hydrogel through sol–gel transition, enabling the facile fabrication of microscale filaments via extrusion [34]. The carbonaceous fiber electrodes derived from agarose-CNTs hydrogel filaments by the Joule heating of nanosoldering exhibit a high rate capability and good cycling performance. Even without pre-existing core fibers, they were confirmed to be mechanically robust enough to operate under highly deformed conditions.

## 2. Materials and Methods

### 2.1. Agarose-CNTs Filaments Preparation

The agarose-CNTs (a-CNT) composite filament was fabricated using the procedure described elsewhere with minor modifications [11]. A total of 2.0 g of a 3 wt% suspension of multiwalled CNTs (MWCNT, Nanostructured and Amorphous Materials, Inc., Houston, TX, USA) dispersed in water was heated to ~100 °C under constant stirring for 5 min. A total of 40 mg of agarose (Acros) was added to the MWCNT suspension and the mixture was stirred for 30 min at ~100 °C. The hot mixture was then transferred to a 5 mL syringe equipped with a needle. The hydrogel filament was formed by syringe pumped extrusion through tubes with 0.5 mm diameter orifices (Tygon; Saint-Gobain Corp., Paris, France) The resulting filaments were dried at room temperature for 12 h, forming the a-CNT composite fibers.

### 2.2. Amorphous Carbon-CNTs Fibers Fabrication

The a-CNT filaments were converted into amorphous carbon-CNTs fibers (aC-CNTs) using the nanosoldering-induced Joule heating process described in a previous report [33]. For nanosoldering, individual a-CNT fibers were loaded onto an electrically insulating substrate. Four different points of a fiber, separated by the same distance, were connected by copper wires using silver paste. The sample was transferred into a reaction chamber, which was subsequently evacuated down to ~1.3 × 10^−8^ Pa to avoid any oxidation during carbonization. Through electrical feedthrough, an initial voltage of 5 V was applied to two outer points and ramped up to 140 V in 5 V increments. The current was recorded, with probes at two inner points, at each voltage.

### 2.3. Fiber SCs (fSCs) Fabrication

A typical procedure to fabricate the PVA/H_3_PO_4_ solid electrolyte is as below. A total of 1.0 g of PVA (Mw~95,000 g mol^−1^, Acros, Carlsbad, CA, USA) was dissolved in 15 mL of Type 1 water at 90 °C, and the resulting solution was stirred vigorously until becoming transparent. After cooling down to room temperature, 0.8 g of H_3_PO_4_ (85 wt% aqueous solution, Aldrich, St. Louis, MO, USA) was added to the solution, which was then stirred overnight to obtain a homogeneous solution. Two aC-CNTs fibers were mounted onto a polyethylene terephthalate (PET) substrate in a symmetrical, parallel configuration. The electrolyte solution was applied to cover the same length of two fibers. The device was left to dry overnight at room temperature, resulting in the formation of the solid electrolyte. For more accurate measurements, the fiber edges not covered by the electrolyte were connected to metal wires using silver paste.

### 2.4. Characterization

The morphologies of fiber samples were investigated using a Hitachi S-4800 (Japan) scanning electron microscope (SEM) at an accelerating voltage of 10 kV. Optical images were taken by a stereo microscope (Amscope ZM-4TNZ, Microscope Central, Feasterville, PA, USA) equipped with a digital camera (EOS Rebel T3, Canon, Japan). Raman spectra were acquired using a Nanophoton Raman-11 system (Japan) with a 532 nm excitation wavelength. Electrical conductivity measurements were conducted using a Keithley 4200 semiconductor analyzer system. Thermogravimetric analysis (TGA) was performed using a TA Instruments Q600 (USA) over the temperature range from room temperature to 800 °C at a heating rate of 10 °C min^−1^ in air. Electrochemical measurements, including cyclic voltammetry (CV), galvanostatic charge and discharge (GCD) cycling, and electrochemical impedance spectroscopy (EIS), were conducted on a Biologic VMP3 multichannel potentiostat (France). EIS measurement was carried out over the frequency (*f*) range of 10^6^–10^−2^ Hz with an AC voltage amplitude of 10 mV. Using the GCD curves, the volumetric capacitances of one electrode (*C_e_*) were calculated based on the following equation [9,35],

*C_e_* = 4*I*/[(∆*E*/∆*t*)*V*],

where *I* is the current applied, ∆*E*/∆*t* is the slope after an initial ohmic voltage drop (=*IR* where *R* is the internal resistance) of the discharge curve, and *V* is the volume of two electrodes (~1.4 × 10^−4^ cm^3^ in general).

## 3. Results and Discussion

**Figure 1:**
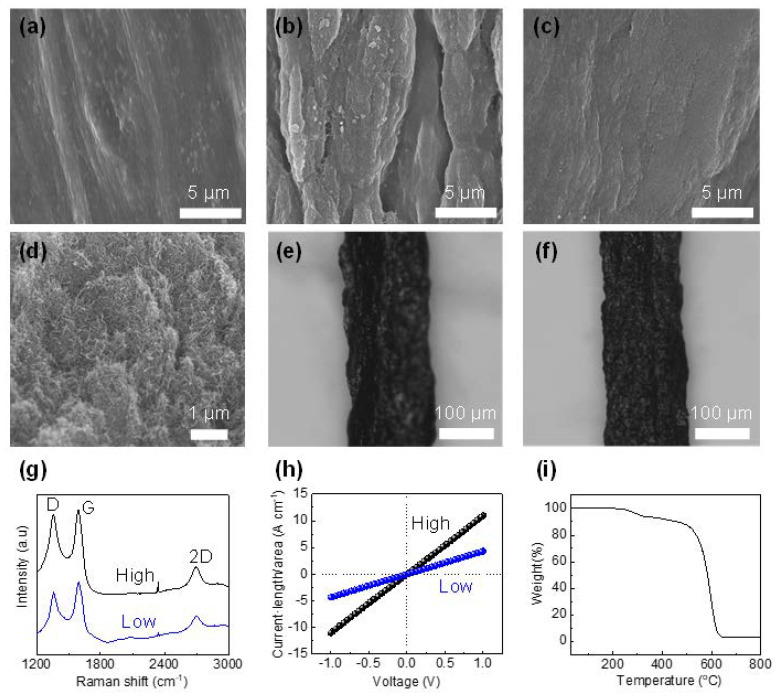
SEM images of (**a**) agarose-CNTs and aC-CNTs nanosoldered at (**b**) low and (**c**) high currents. (**d**) Cross-section SEM images of aC-CNTs nanosoldered at a high current. Optical microscope images of aC-CNTs nanosoldered at (**e**) low and (**f**) high currents. (**g**) Raman spectra and (**h**) current–voltage plots of two nanosoldered samples. (**i**) TGA curve of aC-CNTs nanosoldered at a high current in air.

Figure 1a shows an agarose-CNTs (a-CNT) composite filament with a smooth surface. The agarose and CNTs are homogeneously mixed in the composite, as we indicate in our previous study. This indicates that agarose is a good medium for CNT dispersion [11,34]. In the aC-CNTs fiber, produced by nanosoldering at a low current (16.8 mA) (Figure 1b), an increase in the roughness and agglomeration of components is observed, which could be ascribed to the decomposition and volume change of agarose by Joule heating [33]. By contrast, the aC-CNTs fiber fabricated at a high current (29.1 mA) (Figure 1c) exhibits a smoother surface, relative to the low-current sample, and no agglomeration. This is similar to what is observed in a previous report on the PVA-CNTs fibers [33]. The reason for this is not clear yet, but perhaps a higher current could provide enough driving force for CNTs to spatially reorganize, filling the voids generated by Joule heating. A cross-sectional image (Figure 1d) of the high-current fiber sample presents that CNTs are uniformly dispersed inside the carbon matrix and aligned along an axial direction while preserving the original morphologies. This implies that the high current does not damage the CNTs. Figure 1e,f show optical microscope images of the low-current and high-current-induced samples, respectively. Both fibers are ~150 µm in diameter, which is an approximately four-fold shrinkage from the 0.5 mm diameter of the fiber drawn from the tube. A similar behavior was observed in dried agarose-CNT fibers [11]. Apparently, the high current sample has a smoother surface relative to the low-current one, which is consistent with the SEM observations.

To investigate the impact of the current level on the phase and crystallinity, Raman spectroscopy measurements were performed. As shown in Figure 1g, both nanosoldered samples exhibit three major peaks at 1355, 1592, and 2697 cm^−1^, corresponding to the D mode (sp^3^-based defects), G mode (sp^2^-hybridized graphitic), and 2D mode (second-order D mode) of typical carbonaceous materials [36]. Two samples show the difference in the full width at half maximum (FWHM) of the peaks. For example, the FWHM of the G peak becomes narrower as the nanosoldering current increases: 65 cm^−1^ for the low-current fiber and 61 cm^−1^ for the high current fiber. This indicates that a higher current produces less disordered carbon [37]. A similar trend is also observed in conventional carbonization; graphitic carbons gradually evolve with increasing temperature [37]. Figure 1h shows that the electrical conductivity of the high-current sample (10.9 S cm^−1^) is enhanced by a factor of 2.5 relative to the low-current sample (4.3 S cm^−1^). This may be because electrical connectivity among the CNTs of the high-current sample is highly enhanced compared to the low current sample, as confirmed in the morphologies above (Figure 1b,c). Since the aC-CNTs fiber is composed of amorphous carbon and carbon nanotubes after Joule heating, it shows a typical TGA curve of carbonaceous materials in air (Figure 1i). The weight drops suddenly, starting from ~550 °C, and becomes nearly zero at ~600 °C, indicating full combustion.

**Figure 2:**
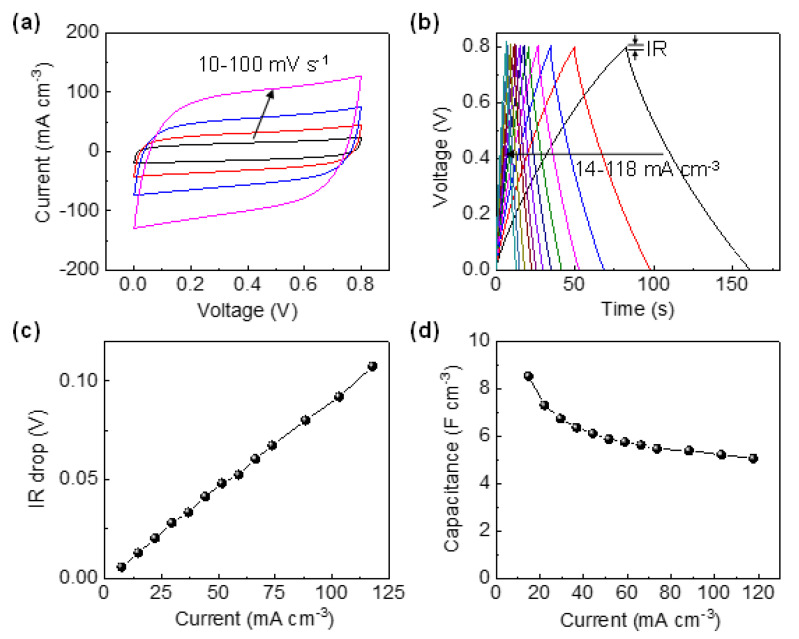
(**a**) CV curves of the aC-CNTs fSCs at scan rates ranging from 10 to 100 mV s^−1^. (**b**) GCD profiles of aC-CNTs fSCs at different current densities ranging from 14 to 118 mA cm^−3^. (**c**) IR drop of aC-CNTs fSCs as a function of current and (**d**) rate-dependent capacitance obtained from GCD curves.

Since the difference in the electrical conductivity between two nanosoldered samples is modest, the electrochemical performances of the high-current sample only (aC-CNTs hereafter) were examined. The fSCs were assembled in a configuration of two parallel fibers separated by the PVA/H_3_PO_4_ solid electrolyte on a flexible PET substrate. The electrochemical properties of the fSCs were evaluated by two-electrode cyclic voltammetry (CV) and galvanostatic charge and discharge (GCD) measurements over the voltage window of 0 to 0.8 V. Figure 2a presents the CV curves at different scan rates ranging from 10 to 100 mV s^−1^. At all scan rates, the CV curves exhibit a nearly rectangular shape, indicating that an electrical double layer effectively forms at the electrode/electrolyte interface and charge propagates smoothly across the electrodes [9,17]. The rate-dependent electrochemical properties of the fSCs were examined by conducting GCD measurements at different current densities ranging from 14 to 118 mA cm^−3^ (Figure 2b). The GCD voltage profiles commonly exhibit a symmetrical triangular shape during charge–discharge processes, a typical behavior of an ideal supercapacitor [38]. This agrees with the CV results. The IR drop, the voltage difference between the initial two points of a galvanostatic discharge curve, was plotted against the current density, as shown in Figure 2c.

The IR drop value linearly increases with the increasing current density. The slope of the plot (9.03 × 10^−4^ V cm^3^ mA^−1^) divided by the electrode volume gives the SCs internal resistance (*R_i_*) of ~3.3 kΩ; *R_i_* is given by *V*/2*I*, where *V* is the IR drop and *I* is the current applied. The rate-dependent volumetric capacitances of one electrode were obtained from the GCD profiles. Figure 2d shows that the aC-CNTs fiber SCs deliver the volumetric capacitance of 8.5 F cm^−3^ at the smallest current density applied (14 mA cm^−3^) and retain 79% of this value when doubled (29 mA cm^−3^). Even at 118 mA cm^−3^, the capacitance still remains at 60%, indicating the high rate capability. At a current density of ~35 mA cm^−3^, the aC-CNTs fiber electrodes (6.4 F cm^−3^) exhibit an improved volumetric specific capacitance (one electrode basis) by a factor of ~3 relative to the a-CNT fiber electrodes (2.4 F cm^−3^), as reported in a previous work [11]. One reason for this may be that, in the aC-CNTs electrodes, the electrical connectivity among the CNTs is well established through the surrounding conductive carbon matrix, unlike the a-CNT electrodes, where the CNTs are linked together in the agarose matrix with no electrical conduction [11].

**Figure 3:**
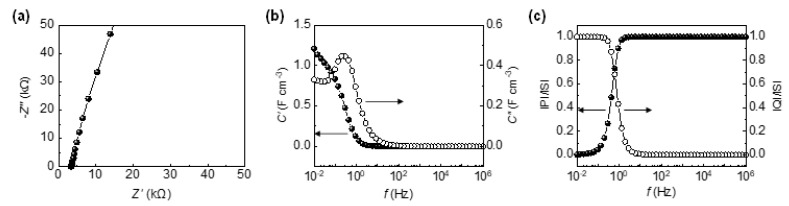
(**a**) Nyquist plot of aC-CNTs fSCs over the frequency range of 10^6^–10^−2^ Hz with a sinusoidal voltage of 10 mV. (**b**) Real (*C’*) and imaginary (*C”*) parts of capacitance of aC-CNTs fSCs as a function of frequency. (**c**) Normalized active (|*P*|/|*S*|) and reactive (|*Q*|/|*S*|) powers of aC-CNTs fSCs as a function of frequency.

To investigate the electrode kinetics in more detail, an electrochemical impedance spectroscopy (EIS) measurement was performed on two-electrode SCs over the frequency range of 10^6^–10^−2^ Hz. The Nyquist plot in Figure 3a is composed of the negative imaginary part ($-Z^{\prime \prime }$) vs. the real part ($Z^{\prime }$) of the complex-plane impedance collected at different AC frequencies. The intercept on the $Z^{\prime }$ axis indicates the equivalent series resistance (ESR) of the aC-CNTs fSCs that is the serial combination of the resistances of electrodes, electrolytes, current collectors, and electrode/current collector interfaces [39]. The ESR of the SCs (~3.4 kΩ) is about two times smaller than that of the a-CNT fSCs (~7.0 kΩ) [11], probably because of the improved electrical connectivity among the CNTs. This supports the rate performance result previously mentioned. We note that both aC-CNTs and a-CNT fSCs employ the same solid electrolyte and similar fiber dimensions [11]. The ESR value is in good agreement with the internal resistance (~3.3 kΩ) determined from the IR drop. In the Nyquist plot, following the intercept the semicircle is not observed at the high frequency range, indicating that facile charge transfer occurs at the electrode/electrolyte interfaces [40]. In the middle frequency range, a straight line with a 45° slope is absent, indicating that Warburg-type diffusion does not occur. One possible reason for this may be that the diffusion kinetics of electrolyte ions into the electrodes is fast enough, probably due to the interconnected ion channel and uniform ion distribution on the surface [12]. A nearly vertical slope in the mid-to-low frequency range is a typical sign of a nearly pure capacitor. This agrees with the shapes of the CV curves and the GCD profiles.

The complex form of cell capacitance includes the information on the charge–discharge rate capability of a SC and is given from the frequency-dependent impedance Z(ω) using the following equations [35]:

$$\begin{array}{c} Z(\omega )=1/j\omega C(\omega ),\;Z(\omega )=Z^{\prime }(\;\omega )+jZ^{\prime \prime }(\omega ),\\ C(\omega )=C^{\prime }(\;\omega )-jC^{\prime \prime }(\omega ),\\ C^{\prime }(\omega )=\frac{-Z^{\prime \prime }(\omega )}{\omega {\left|Z(\omega )\right|}^{2}},\;C^{\prime \prime }(\omega )=\frac{Z^{\prime }(\omega )}{\omega {\left|Z(\omega )\right|}^{2}}, \end{array}$$

where *ω* is the angular frequency (=2*πf*) and *C’*(*ω*) and *C”*(*ω*) are the real part and the imaginary part of the complex impedance *C*(*ω*), respectively. Figure 3b shows that, at high frequencies, *C’*(*ω*) asymptotically reaches zero because of the resistive behavior at short times. As the frequency decreases, *C’*(*ω*) demonstrates a frequency-dependent response and reaches 1.2 F cm^−3^ at the lowest frequency measured (10^−2^ Hz). This value agrees with the volumetric capacitance of the cell (5.1/4 = 1.3 F cm^−3^) [9,35] obtained from the GCD measurement at a current density of 118 mA cm^−3^. The imaginary part, *C”*(*ω*) in Figure 3b, reveals the energy dissipation of SC, and has a more explicit form of resistor to capacitor transition as a function of the frequency. The peak maximum of *C”*(*ω*) (or the half maximum of *C’*(*ω*)) appears at a frequency, *f*_0_, where the resistive and capacitive behaviors are equal [11,35]. The characteristic relaxation time constant (*τ*_0_), which reveals the response kinetics of a supercapacitor, is determined by *τ*_0_ *= 1/f*_0_. For the aC-CNTs fSCs, *f*_0_ = 0.4 Hz and *τ*_0_ = 2.5 s. This time constant is four times smaller than that (10 s) of the a-CNT fSCs [11], reflecting that the SCs charge and discharge much faster because of their negligible interfacial charge transfer resistance, facile ion diffusion kinetics, and highly conductive electrodes.

To examine the energy storage and dissipation characteristics, a complex power analysis was performed using the complex capacitance [35]. The complex power, *S*(*ω*), has a form of:

$$\begin{array}{c} S(\omega )=P(\omega )+jQ(\omega ),\\ P(\;\omega )=\omega C^{\prime \prime }(\omega ){\left|\Delta V_{rms}\right|}^{2},\\ Q(\;\omega )=-\omega C^{\prime }(\omega ){\left|\Delta V_{rms}\right|}^{2}, \end{array}$$

where *P*(*ω*) and *Q*(*ω*) denote the real and the imaginary parts and $∆V_{rms}\;=\;∆V_{max}/\sqrt{2}$ (∆*V_max_*: maximum amplitude of applied sinusoidal voltage). *P*(*ω*) refers to the active power and Q(ω) the reactive power. Figure 3c presents the normalized real part |*P*|/|*S*| and imaginary part |*Q*|/|*S|* as a function of frequency. The |*P*|/|*S*| is unity at high frequencies but rapidly decreases to zero as the frequency decreases, and the |*Q*|/|*S|* shows the opposite trend, indicating that the fSCs dissipate 100% energy at a high frequency whereas they store energy at a low frequency. The results confirm that the aC-CNTs fSCs respond as a pure resistor at a fast switching rate and a pure capacitor at a slow switching rate. The characteristic relaxation time constant (*τ*_0_) is determined from the frequency (*f_o_*), where |*P*|/|*S*| = |*Q*|/|*S*| = 1/$\sqrt{2}$ at the phase angle of 45° [35]: *f*_0_ = 0.6 Hz, *τ*_0_ = 1.7 sec. This agrees with the result from the complex capacitance analysis.

**Figure 4:**
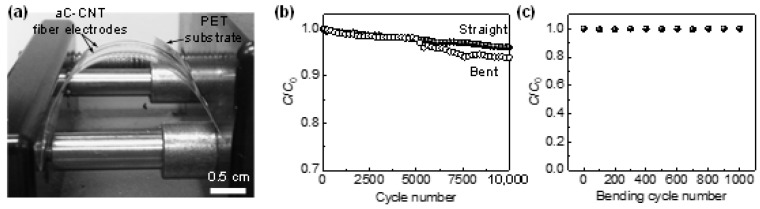
(**a**) A photograph of a setup for the flexibility test of aC-CNTs fSCs. (**b**) Capacitance retention of aC-CNTs fSCs over 10,000 charge–discharge cycles at 14 mA cm^−3^ in straight and bent states. (**c**) Capacitance stability of aC-CNTs fSCs over 1000 bending-straightening cycles.

For potential applications in curved surfaces or in complex geometry, the aC-CNTs fSCs need to demonstrate the high and stable storage performances when deformed. As shown in Figure 4a, the fSCs integrated on the PET substrate were bent with a radius of curvature of ~12 mm (90° bend), which is determined to be the minimum value for stable charge and discharge, and their galvanostatic cycling stability was evaluated. Figure 4b demonstrates that, in their bent state, the electrodes retain 94% of the initial capacitance for at least 10,000 charge/discharge cycles at a constant current of 14 mA cm^−3^, which is nearly identical to the straight (undeformed) sample after the same cycles (96% capacitance retention). This indicates that the aC-CNTs fiber electrodes still preserve good electronic and ionic pathways even when deformed. Furthermore, the SCs preserve nearly 100% of the initial capacitance after 1000 cycles of bending-straightening test, with a radius of bending curvature of ~12 mm (Figure 4c). The overall results imply that the aC-CNTs fiber electrodes barely suffer from any significant mechanical damage and electrical disconnection when subjected to mechanical stress.

## 4. Conclusions

In conclusion, we report the fabrication and characterization of flexible microscale fSCs electrodes consisting of CNTs embedded in an amorphous carbon matrix, enabled by the extrusion of agarose-CNTs composite hydrogels followed by nanosoldering. The aC-CNTs fSC electrodes demonstrate a high volumetric capacitance and rate performance, which is attributed to their fast response kinetics. They also exhibit a long cycle life when deformed and a stable electrochemical performance under repeated bending-straightening cycles. The fabrication process could be generally applicable to convert any fibrous hydrogel-embedded hydrophilic materials into carbon-based composite fibers. We expect that this work will have broad impacts on manufacturing advanced fiber materials for applications in energy storage devices and sensors requiring flexibility and wearability.
